# A randomized trial to investigate the efficacy and safety of insulin glargine in hyperglycemic acute stroke patients receiving intensive care

**DOI:** 10.1038/s41598-021-91036-2

**Published:** 2021-06-01

**Authors:** Sung-Chun Tang, Shyang-Rong Shih, Shin-Yi Lin, Chih-Hao Chen, Shin-Joe Yeh, Li-Kai Tsai, Wei-Shiung Yang, Jiann-Shing Jeng

**Affiliations:** 1 Stroke Center and Department of Neurology, National Taiwan University Hospital, No. 7 Chung-Shan South Road, Taipei, 10055 Taiwan; 2 Department of Internal Medicine, National Taiwan University Hospital, No. 7 Chung-Shan South Road, Taipei, 10055 Taiwan; 3 Department of Pharmacy, National Taiwan University Hospital, Taipei, Taiwan; 4 School of Pharmacy, College of Medicine, National Taiwan University, Taipei, Taiwan; 5 Graduate Institute of Clinical Medicine, National Taiwan University, Taipei, Taiwan

**Keywords:** Neurological disorders, Stroke, Diabetes complications, Type 2 diabetes

## Abstract

This pilot, randomized, open-label controlled study compared the basal–bolus regimens of insulin glargine (IG) and neutral protamine Hagedorn (NPH) insulin in stroke patients with hyperglycemia receiving intensive care. The study recruited acute stroke patients requiring intensive care within 72 h (h) of onset and had blood glucose > 200 mg/dL. 50 patients received IG (n = 26) or NPH (n = 24) with added short-acting prandial regular insulin over a 72-h period. The primary end point was the percentage of glucose within 80–180 mg/dL assessed through continuous glucose monitoring. The baseline characteristics were comparable, except the IG had higher glucose pre-randomization than the NPH (290.69 ± 82.31 vs. 246.04 ± 41.76 mg/dL, *P* = 0.021). The percentage of time with glucose between 80 and 180 mg/dL was 45.88 ± 27.04% in the IG and 53.56 ± 22.89% in the NPH (*P* = 0.341) and the percentage of glucose reduction was 31.47 ± 17.52% in the IG and 27.28 ± 14.56% in the NPH (*P* = 0.374). The percentage of time with glucose < 60 mg/dL was 0.14 ± 0.49% in the IG and 0.47 ± 1.74% in the NPH. Poststroke outcomes were not significantly different. In conclusion, IG is safe and equally effective as an NPH-based basal-bolus regimen for acute stroke patients with hyperglycemia receiving intensive care.

**Trial registration** ClinicalTrials.gov, NCT02607943. Registered 18/11/2015, https://clinicaltrials.gov/ct2/show/NCT02607943.

## Introduction

Diabetes mellitus is a strong independent risk factor for the development of stroke^1^. It is known that stroke initiates several mechanisms that lead to hyperglycemia, such as increased innate immunity and stimulation of the hypothalamic–pituitary–adrenal axis^2^. Therefore, patients with acute stroke, especially those with a history of diabetes mellitus, frequently have hyperglycemia^3, 4^. Notably, numerous studies have shown that persistent poststroke hyperglycemia is closely associated with stroke-in-evolution, symptomatic hemorrhagic transformation, hematoma expansion, and unfavorable functional outcomes^5–8^.

The Guidelines for the Early Management of Patients with Acute Ischemic Stroke from the American Heart Association and the American Stroke Association suggest that the blood glucose level should be maintained between 140 and 180 mg/dL, in accordance with the current American Diabetes Association recommendation^9, 10^. Currently, multiple protocols of subcutaneous and intravenous insulin treatments are used to ameliorate hyperglycemia in patients with different diseases during hospitalization^5^. Continuous intravenous insulin infusion achieves rapid correction of hyperglycemia and is effective in maintaining glucose within a strict predetermined range^11^. However, a recent trial suggested that for patients with acute ischemic stroke and hyperglycemia, aggressive intravenous insulin infusion has a significantly higher chance of inducing hypoglycemia without absolute clinical benefits compared with subcutaneous insulin injection^12^.

Neutral protamine Hagedorn (NPH) insulin is widely used for the treatment of hyperglycemia during hospitalization. The duration of its effects is approximately 12 h, and it exhibits a peak in its time-action profile at 5–7 h. These properties mean that NPH does not fully mimic the physiological secretion of basal insulin. By contrast, long-acting insulin analogs such as insulin glargine (IG) create a subcutaneous depot after injection, exhibit no pronounced peak after administration, and last for 24 h. They are currently preferred for glycemic control of diabetes^13^. Studies have demonstrated that IG is safe in various clinical situations, including for both non-critically and critically ill hospitalized patients^14–18^. However, evidence regarding the use of IG in patients with acute stroke and hyperglycemia is limited. The objective of this randomized controlled study was to determine the efficacy and safety of early initiation of subcutaneous once-daily IG compared with standard NPH regimens to achieve proper glycemic control in patients with acute stroke and hyperglycemia admitted to the intensive care unit (ICU).

## Methods

This is a randomized, open-label, pilot clinical trial. Patients with acute stroke admitted to the stroke ICU of National Taiwan University Hospital within 72 h of onset (onset defined as the last known time at which the patient was well) were screened. The entry criteria for admittance to the stroke ICU included ischemic stroke with thrombolytic therapy or endovascular treatment, intracerebral hemorrhage with aggressive blood pressure control treatment, severe neurological deficits [e.g., a National Institute Health Stroke Scale score (NIHSS) higher than 8], stroke-in-evolution, or other medical conditions requiring intensive care^19, 20^. Patients who met the inclusion criteria including age ≥ 20 years, and capillary blood glucose > 200 mg/dL after admission were enrolled. Patients were excluded if they had any of the following conditions: autoimmune disease, human immunodeficiency virus infection, sepsis, pregnancy, treatment with corticosteroids or vasopressors, end-stage renal disease requiring dialysis, type I diabetes mellitus, or hypersensitivity to any insulin products.

The diagnosis of stroke was confirmed through diffusion-weighted magnetic resonance imaging of the head or repeated computed tomography scanning. Patients with ischemic stroke were further classified into 5 major subtypes according to the Trial of ORG 10,172 in Acute Stroke Treatment criteria^21^. A detailed history of each patient’s clinical presentation, vascular risk factors, and comorbidity was obtained. Body mass index was calculated as weight divided by the square of height in meters. Stroke severity on admission was assessed using the NIHSS by the consulting neurologists. Mortality and functional outcome 3 months poststroke were assessed using the modified Rankin scale. Acute stroke care for both groups followed the latest guidelines from the American Heart Association and the American Stroke Association^9, 22^.

### Randomization

Patients were assigned to the IG group or the NPH group through computer-generated randomization. Allocation was concealed by the use of opaque, consecutively numbered envelopes. Treatment allocation was 1:1.

### Insulin regimen

Two endocrinologist (Dr. Shih and Yang) designed the insulin regimens and managed the insulin titration protocols. All participants received only insulin for glycemic control during the intervention period, which was the first 72 h after study enrollment. Administration of oral hypoglycemic agents and noninsulin injections, regardless of whether they were used before stroke, were stopped. The insulin regimens in both groups are described as follows. A basal–bolus regimen of IG or NPH was used for glycemic control. All patients received regular insulin before meals as a correctional component. During the period when food was prohibited (nothing-by-mouth status), IG was administered because it does not demonstrate a pronounced peak. NPH was withheld to prevent hypoglycemia.

The total daily insulin dose (TDD) was based on weight^11^. For most patients, the TDD was 0.6 U/kg/day. For patients anticipated to be insulin sensitive, such as those aged over 80 years or those whose creatinine clearance rate was below 30 mL/min, the TDD was empirically reduced to 0.5 U/kg/day. The TDD of patients receiving insulin therapy before admission was based on their outpatient dose and modified by an endocrinologist. For the IG group, 50% of the TDD was basal insulin and the other 50% was evenly administered before each meal. For the NPH group, 25% of the TDD was basal insulin, and similarly, the remaining 75% was administered evenly before meals. After the regimens were administered, an endocrinologist (Dr. Shih) carefully assessed glycemic levels and made daily adjustments to achieve glycemic levels between 80 and 180 mg/dL. Scheduled prandial or basal insulin administration was temporarily stopped during treatment of hypoglycemia (defined as blood glucose < 60 mg/dL), which was performed in accordance with a previously published guideline^23^.

### Blood glucose measurements

Participants’ capillary blood glucose was measured before each meal and once during fasting, at the frequency of every 4–6 h, depending on their eating schedule. In addition, a Medtronic Enlite Glucose Sensor (Medtronic, Northridge, CA, USA) was used to monitor glycemia during the intervention period^24^. Interstitial glucose levels were recorded every 5 min with a detection range of 40–400 mg/dL. Glucose levels from capillary blood and continuous glucose monitoring (CGM) were compared and calibrated at randomization and 1, 3, and 4 h after randomization. The glycemic data from CGM were used in the analyses.

### Study outcomes

The primary efficacy outcome was the percentage of glucose levels within the range of 80–180 mg/dL during the 72-h intervention period. The primary safety outcome was the percentage of time with hypoglycemia (glucose < 60 mg/dL). The secondary clinical outcomes were determined according to whether a favorable outcome was achieved (modified Rankin scale score ≤ 2), whether a poor outcome was achieved (modified Rankin scale score ≥ 4), mortality, and Barthel index scores at 3 months poststroke. The clinical outcomes at 3 months after ischemic stroke were assessed at return appointment or by a telephone interview by our study nurses who were blinded from study subjects’ grouping. The secondary laboratory outcomes were glycemic mean and variability, determined from CGM data. Glycemic variability was calculated through linear and nonlinear analyses using previously described methods. We computed two-time domain measures as standard deviations and the root mean square of successive beat-to-beat differences. The nonlinear analysis of sample entropy was also applied to the glucose data using methods described previously^19, 25, 26^.

### Statistical analysis

This is a pilot study without formal power justification. Sample size calculation was not directly applicable in this trial because the expected effect size of our primary outcome, namely the percentage of time before return to normoglycemia, was yet unknown. Instead, we followed a previously proposed method of enrolling at least 50 participants in a pilot study^27^. The analysis was performed as intention-to-treat to reflect clinical practice in stroke ICUs. Descriptive statistics of baseline characteristics of the study population are expressed as numbers (percentages) and means ± standard deviations. Because the primary efficacy and safety outcomes were nonnormally distributed quantitative variables, the Mann–Whitney U test was used to determine whether the between-group differences in mean percentage of time were significant. Between-group mean differences and 95% confidence intervals of the primary outcomes were also analyzed. The chi-square test or the Fisher’s exact test were applied for categorical secondary outcomes, whereas the Mann–Whitney U test was applied for the continuous variables. In the multivariable analysis for the factors significantly associated with favorable outcomes, a logistic regression method was used to adjust with age, sex, insulin group, and factors with univariate *P* values of < 0.05, including NIHSS at admission, ICH, body weight, and levels of hemoglobin. Statistical analyses were performed using IBM SPSS Statistics for Windows, version 26 (IBM Corp., Armonk, NY, USA).

### Ethics

This study was approved by the Institutional Review Board and the Medical Ethics Committee of National Taiwan University Hospital (Clinicaltrials.gov number: NCT02607943, date of registration: 18/11/2015). In the initial protocol, the study had been proposed to recruit stroke patients within 24 h in multi-centers. But we changed the protocol to recruit patients within 72 h after stroke due to delay in participant enrollment process and modified the study site to single center due to restriction of study funding. The revised protocol has been approved by the NTUH Ethical Committee. All participants provided informed consent or were recruited with informed consent provided by their first-degree relatives. The study was conducted in accordance with the revised version of the 2008 Declaration of Helsinki.

## Results

### Patient characteristics

Between December 2015 and October 2019, 50 patients with stroke were randomized; 26 were assigned to the IG group and 24 were assigned to the NPH group. The study flow diagram was shown in supplementary Fig. 1. Two participants (one in each group) did not have CGM data because of unexpected technical issues. Of the participants, 37 (74%) were admitted with ischemic stroke, and the remainder were admitted with intracerebral hemorrhage. Two patients in the IG group were lost to follow-up at 3 months poststroke. A total of 14 patients (29.2%) had favorable outcomes, and 17 patients (35.4%, including 5 who died) had poor outcomes.

The participant clinical characteristics are listed in Table 1. Baseline profiles were comparable between the groups for age, sex, body mass index, prestroke functional status, stroke type, stroke severity on admission, stroke risk factors, percentage of participants receiving acute reperfusion therapy, and biochemical data on admission. However, creatinine levels were higher in the NPH group than in the IG group (1.21 ± 0.45 vs. 0.97 ± 0.28, *P* = 0.027). In the NPH and IG groups, 8 and 10 patients had a nothing-by-mouth status when insulin regimens were initiated. Most of them started eating within several hours, but 3 patients in the IG group each fasted for 12, 19, and 41.5 h during the intervention period.

**Table 1:** The baseline characteristics of AIS patients.

	NPH insulin (n = 24)	Insulin glargine (n = 26)
Age, year	62.7 ± 16.0	64.7 ± 14.6
Male (N)	16 (66.7)	15 (57.7)
BMI (kg/m^2^)	26.3 ± 4.1	26.5 ± 5.2
Weight (kg)	70.3 ± 15.5	70.5 ± 16.5
History of stroke	9 (26.3)	4 (3.7)
Pre-mRS ≥ 2	5 (20.8)	4 (15.4)
NIHSS at admission	11.5 (6, 17)	13 (8, 21)
Atrial fibrillation	2 (8.3)	3 (11.5)
Diabetes mellitus	23 (95.8)	22 (84.6)
Hypertension	22 (91.7)	19 (84.6)
Hyperlipidemia	9 (37.5)	10 (38.5)
CAD	5 (20.8)	6 (23.1)
**Infarction**	18 (75.0)	19 (73.1)
Cardioembolism	6 (33.3)	6 (31.6)
LAA	7 (38.9)	7 (36.8)
Others	5 (27.8)	6 (31.6)
ICH	6 (25.0)	7 (26.9)
Smoking	7 (29.2)	5 (19.2)
IV rt-PA	5 (20.8)	7 (26.9)
EVT	6 (25.0)	5 (19.2)
TG (mg/dl)	200.08 ± 119.88	190.0 ± 263.67
Total cholesterol (mg/dl)	189.00 ± 52.43	175.88 ± 65.03
LDL (mg/dl)	108.25 ± 31.52	105.96 ± 45.13
Glucose at admission (mg/dl)	317.00 ± 116.85	270.88 ± 99.87
HbA1c (%)	9.84 ± 2.10	9.15 ± 1.94
Creatinine (mg/dl)*	1.21 ± 0.45	0.97 ± 0.28
Hemoglobin (g/dl)	14.30 ± 2.22	14.30 ± 2.11
WBC (K/ul)	9.07 ± 2.85	10.17 ± 3.00
Platelet (k/ul)	238.58 ± 53.87	224.65 ± 61.51
PTT (second)	25.68 ± 2.29	25.61 ± 2.50
INR	0.97 ± 0.15	0.98 ± 0.06

Data was expressed as number (proportion) or mean ± standard deviation.

*NPH* neutral protamine hagedorn insulin, *BMI* body mass index, *mRS* modified Rankin Scale, *NIHSS* National Institute of Health Stroke Scale, *CAD* coronary artery disease, *LAA* large artery atherosclerosis; Others include other determined and undetermined. *ICH* intracerebral hemorrhage, *IV* intravenous, *EVT* endovascular thrombectomy, *WBC* white blood cell, *PTT* partial thromboplastin time, *INR* international normalized ratio, *TG* triglyceride, *LDL* low-density lipoprotein, *HbA1c* glycated hemoglobin.

*Creatinine (mg/dl) is the only one baseline parameter with significant difference between these two groups.

### Effectiveness, safety, and variability

Glycemic levels before randomization were significantly higher in the IG group than in the NPH group (290.69 ± 82.31 vs. 246.04 ± 41.76 mg/dL, *P* = 0.021) (Table 2). The percentage of time with glucose levels between 80 and 180 mg/dL was 45.88 ± 27.04% in the IG and 53.56 ± 22.89% in the NPH (*P* = 0.341) and the percentage of glucose reduction was 31.47 ± 17.52% in the IG and 27.28 ± 14.56% in the NPH (*P* = 0.374).

**Table 2:** Study outcome between treatment groups.

	NPH insulin (n = 24)	Insulin glargine (n = 26)	Mean difference (95% CI)	*P* value
**Primary outcome**				
Pre-randomization (mg/dL)	246.04 ± 41.76	290.69 ± 82.31	44.65 (7.05–82.25)	0.021*
Proportion of time in glucose 80–180 (mg/dL)	53.56 ± 22.89	45.88 ± 27.04	− 7.67 (− 22.30 to 6.95)	0.341
Percentage of glucose reduction (mg/dL)^a^	27.28 ± 14.56	31.47 ± 17.52	4.19 (− 5.21 to 13.60)	0.374
**Secondary outcome (laboratory)**^**b**^				
< 60 mg/dL (number of patients)	3 (12.5%)	2 (7.7%)	NA	0.660
< 60 mg/dL (% of time)	0.47 ± 1.74	0.14 ± 0.49	− 0.33 (− 1.61 to 0.39)	0.361
Mean, mg/dL	175.82 ± 30.16	189.89 ± 32.86	14.07 (− 4.31 to 32.45)	0.130
SD	40.83 ± 11.57	41.11 ± 14.32	0.29 (− 7.32 to 7.89)	0.958
RMSSD	2.99 ± 1.04	3.16 ± 1.06	0.177 (− 0.43 to 0.79)	0.561
Entropy	0.08 ± 0.04	0.07 ± 0.04	− 0.147 (− 0.39 to 0.01)	0.234
**Secondary outcome (clinical)**^**c**^				
mRS ≤ 2, n(%)	8 (33.3)	6 (25.0)	NA	0.752
mRS ≥ 4, n(%)	9 (37.5)	8 (33.3)	NA	1.00
Mortality, n(%)	2 (8.3)	3 (12.5)	NA	1.00
BI Scores, mean ± SD	52.29 ± 37.39	50.42 ± 34.89	− 1.88 (− 22.89–19.14)	0.858

Data was expressed as number (proportion) or mean ± standard deviation.

*NPH* neutral protamine hagedorn insulin, *SD* standard deviation, *RMSSD* root-mean-square of successive beat-to-beat differences, *mRS* modified Rankin scale, *BI* Barthel index, *NA* not available.

^a^Glucose reduction in comparison to pre-randomized glucose level.

^b^n = 24 in Glargine group because two subjects were lost of follow-up.

^c^n = 23 and 25 in two groups, respectively, due to two subjects with unexpected technical issues of the continuous glucose monitor device.

Hypoglycemia, glucose < 60 mg/dL.

For the safety outcome, 3 patients in the NPH group and 2 patients in the IG group had hypoglycemia. The percentage of time with hypoglycemia was very low and comparable between the groups (0.14 ± 0.49% in the IG group vs. 0.47 ± 1.74% in the NPH group, *P* = 0.361). Most hypoglycemic events occurred during fasting (from after dinner on the first day to before breakfast on the second day). All patients were asymptomatic during the hypoglycemic period.

**Figure 1:**
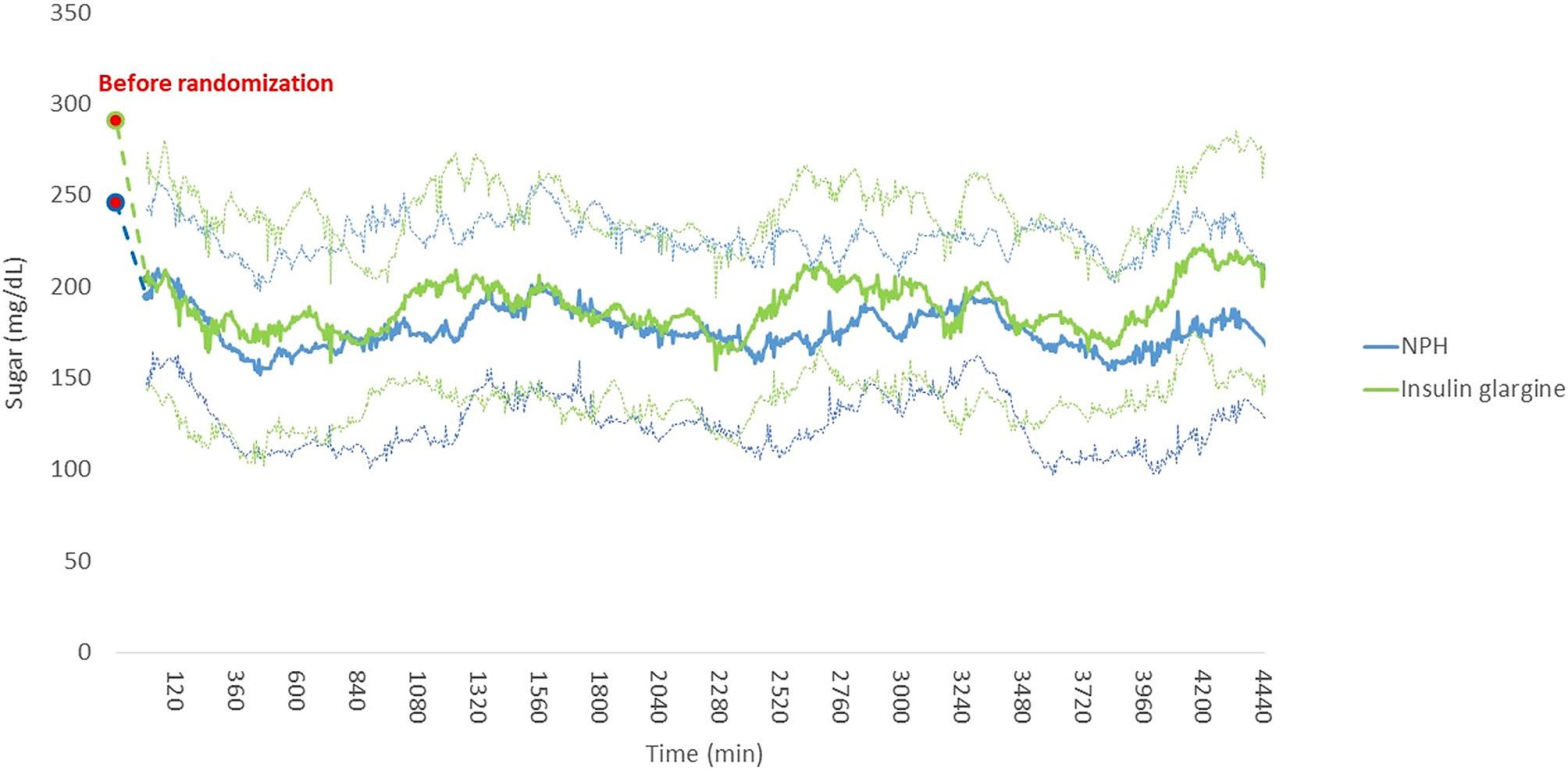
The line graphs of the average and standard deviation of glucose levels at prerandomization and during the 72-h intervention period in insulin glargine and NPH insulin groups.

Figure 1 presents line graphs of the 2 groups’ average and standard deviation of glucose levels at prerandomization and during the 72-h intervention period. The glucose variability is presented as standard deviations. The root mean square of successive beat-to-beat differences, and sample entropy did not differ significantly between the groups (Table 2). The capillary blood glucose of the 2 patients without CGM data, measured every 4 h during the intervention period, also did not differ significantly. All of the secondary clinical outcomes, namely favorable and poor outcomes, mortality, and Barthel index scores 3 months poststroke, were comparable between the groups (Table 2).

### Factors associated with a favorable outcome

As Supplementary Table 1 shows, participants with favorable outcomes were younger, heavier, had lower NIHSS on admission and were more likely to have intracerebral hemorrhage stroke subtype, and had higher levels of hemoglobin compared with those with poor outcomes. None of the glucose-related parameters, including insulin regimen, were associated with significant differences between favorable and poor outcomes. Multivariate analysis showed that only a younger age and lower NIHSS on admission were significantly associated with favorable outcomes (odds ratio = 0.805 and 0.662, 95% confidence interval = 0.651–0.996 and 0.447–0.981, *P* = 0.046 and 0.040, respectively), as presented in Table 3.

**Table 3:** Multivariable analysis for factors predicting AIS patients with good outcome.

Covariate	β estimate	*P*	odds ratio	95% CI
Sex (male)	− 0.571	0.765	0.565	0.013–23.864
Age (per year)	− 0.216	0.046*	0.805	0.651–0.996
NIHSS at admission	− 0.412	0.040*	0.662	0.447–0.981
ICH	0.872	0.482	2.391	0.211–27.148
Weight (kg)	− 0.136	0.139	0.874	0.731–1.045
Hemoglobin (g/dl)	0.736	0.259	2.087	0.582–7.493
Insulin Glargine	− 1.252	0.361	0.286	0.020–4191

*NIHSS* National Institute of Health Stroke Scale, *ICH* intracerebral hemorrhage.

*Statistical significance.

## Discussion

This pilot study was designed to assess the safety and efficacy of using IG as compared to NPH in the management of hyperglycemia in patients with acute stroke. To the best of our knowledge, this is the first randomized clinical trial to investigate the feasibility of early initiation of long-acting IG in patients with acute stroke receiving intensive care. Our results indicate that IG is equally safe and as effective as NPH for use to achieve glycemic control in patients with stroke in an ICU setting. Moreover, no significant between-group differences in outcome parameters were observed.

Whether glucose should be controlled intensively and be consistently maintained at a low level in the acute phase of stroke to prevent hyperglycemia-related secondary brain injuries remains subject to debate. Several clinical trials have compared intensive intravenous insulin and standard care in patients with poststroke hyperglycemia^28–30^. Most trials have reported the efficacy of intensive intravenous insulin infusion in strict glycemic control but also increased risk of hypoglycemic complications. The overall benefit of intravenous insulin infusion for poststroke outcomes is unclear, primarily because of the small sample sizes of relevant studies^31^.

The results of the Stroke Hyperglycemia Insulin Network Effort randomized clinical trial were recently published^12^. The purpose of the trial was to determine and compare the efficacy of intensive and standard treatment of hyperglycemia in patients with acute ischemic stroke. The study comprised 1151 participants randomly assigned to receive continuous intravenous insulin infusion through use of a computerized decision support tool to achieve blood glucose levels of 80–130 mg/dL or to receive subcutaneous insulin infusion on a sliding scale to achieve blood glucose levels of 80–179 mg/dL for up to 72 h. The results showed that the percentages of patients with favorable functional outcomes were comparable between the intensive and standard treatment groups (20.5% and 21.6%, respectively), but the incidence of early discontinuation of treatment due to hypoglycemia or other adverse effects was higher in the intensive group than in the standard group (11.2% vs. 3.2%). These findings do not support the routine use of intensive glycemic control in patients with acute ischemic stroke and hyperglycemia.

Notably, glycemic control in the standard treatment group of the Stroke Hyperglycemia Insulin Network Effort trial involved only a sliding scale of subcutaneous rapid-acting insulin administered every 6 h. Despite its convenience and simplicity, use of only sliding-scale insulin therapy in inpatient settings has been demonstrated to be worse for glycemic control compared with basal–bolus therapy and is strongly discouraged in the current American Diabetes Association guidelines^23^. Sliding-scale insulin therapy is reactive, does not enable adjustments to be made according to the carbohydrate content of meals, and most importantly, does not mimic the physiological delivery of insulin. By contrast, basal–bolus insulin therapy more closely mimics physiological insulin secretion. Administering daily long-acting insulin on top of prandial rapid-acting insulin reduces not only mean daily glucose levels but also glycemic fluctuation. An insulin regimen comprising basal and correction components is the preferred treatment for noncritically ill hospitalized patients with nothing-by-mouth status^32, 33^. For patients with a stable oral intake, a correction component should be added^23^. However, basal–bolus regimens with long-acting IG have not been tested in patients with acute stroke in intensive care.

Several studies have compared IG- and NPH-based regimens in hospitalized patients. Most of them have reported comparable glycemic control between the 2 types of insulin^34–36^. However, one investigation demonstrated a higher incidence of hypoglycemic events in the NPH group^34^, and another reported that the daily insulin requirement was lower in the NPH group^35^. In our study, the primary and secondary endpoints were mostly comparable between the groups. The proportion of patients with hypoglycemia was very low and also comparable between the groups. Notably, most episodes of hypoglycemia occurred during fasting (from after dinner on the first day to before breakfast on the second day). In addition, one patient in the IG group extended nothing-by-mouth status after the intervention and experienced hypoglycemia. However, none of the patients with hypoglycemia developed clinical signs or complications. Physicians should be more aware of patients’ glycemic levels during long fasting periods and promptly treat hypoglycemia should it occur.

Although the percentage of time for glucose between 80 and 180 mg/dL was lower in the IG group than in the NPH group, the percentage of glucose reduction compared with prerandomization levels was higher in the IG group than in the NPH group. This suggests that the insulin regimens may be equally effective and that the mentioned differences could be attributable to the different baseline glucose levels and relatively short intervention period. In addition, 90% of the participants had prestroke diabetes, and the average glycated hemoglobin and admission blood glucose levels were higher than 9% and 250 mg/dL, respectively. The starting dose we selected may have been too conservative for these patients. The TDD of the 2 groups was comparable throughout the intervention period (Supplementary Table 2), but the total daily basal insulin dose was significantly higher in the IG group (27.5 ± 11.3 U/kg in the NPH group versus 57.2 ± 19.4 U/kg in the IG group, *P* < 0.001). The data correlated well with our study design; 50% and 25% of the TDD was basal insulin in the IG group and the NPH group, respectively.

This study has several strengths. It is the first to compare guideline-recommended basal–bolus insulin regimens for the treatment of acute stroke of moderate to high severity requiring critical care. The participants in our study received the optimal treatment for hyperglycemia rather than intensive insulin infusion or regular sliding-scale insulin therapy. In addition, to determine glycemic variability, we performed CGM subcutaneously, ensuring continual delivery of glycemic data (every 5 min) during the intervention period. Thus, the effectiveness and safety of both insulin regimens were comprehensively evaluated.

This study also has some limitations. First, the small sample size and proportion of patients with the heterogeneous stroke subtype as well as the significant difference of glucose levels at pre-randomization in two groups may have limited the power of detection of between-group statistical differences in the endpoints. So all outcomes, including P values, should be cautiously and descriptively interpreted. Second, the intervention period (< 72 h) may have been too short to observe the benefits of glycemic control in stroke outcomes. Third, the study subjects were patients with acute stroke hospitalized in ICU with certain characteristics such as onset within 72 h. Besides, most of them had previous history of diabetes, thus, selection bias may exist, and the results might not be generalizable in other cohorts or in acute stroke patients. Forth, during the period when food was prohibited, IG was administered routinely but not NPH, which may be conceived as a potential performance bias. Nevertheless, the successful application of IG-based basal–bolus regimens can serve as a reference for future studies with larger sample sizes and more specific groups of patients with acute stroke, which would broaden the generalizability of our findings.

## Conclusions

This study demonstrates that IG-based basal–bolus regimens are safe and feasible for patients with acute stroke and hyperglycemia requiring intensive care. Early administration of basal–bolus insulin regimen in the glycemic management of hospitalized patients with stroke, rather than dependence on sliding-scale insulin, should be promoted.

## Supplementary information

Supplementary Figure S1.

Supplementary Tables.

## Data Availability

The datasets generated during and/or analyzed during the current study are available from the corresponding author on reasonable request.
