# Supplementary material for: A randomized trial to investigate the efficacy and safety of insulin glargine in hyperglycemic acute stroke patients receiving intensive care
Source: Sci Rep. 2021 Jun 1;11:11523. doi: 10.1038/s41598-021-91036-2 (PMC8169927; doi:10.1038/s41598-021-91036-2)
Supplement: Supplementary file 1 — Supplementary Figure S1. [file 41598_2021_91036_MOESM1_ESM.doc]

Assessed for eligibility (n=953)

Excluded (n=903)

♦  Not meeting inclusion criteria (n=830)

♦  Participating other trials (n= 3 )

♦  Dialysis (n=2)

♦  Hemodynamics unstable (n=8)

♦  Other reasons (n= 60 )

Analysed (n= 26)
♦ Excluded from analysis (n= 0)

Lost to follow-up (n= 0)

Discontinued intervention (n= 0)

Allocated to insulin Glargine (n= 26)

♦ Received allocated intervention (n= 26)

♦ Did not receive allocated intervention (n= 0)

Lost to follow-up (n= 0)

Discontinued intervention (n= 0)

Allocated to NPH Insulin (n= 24)

 Received allocated intervention (n= 24)

 Did not receive allocated intervention (n= 0)

Analysed (n= 24)
♦ Excluded from analysis (n= 0)

Randomized (n=50)

**Supplementary figure legend:**

The flowchart of the trial.
