# Supplementary material for: A randomized trial to investigate the efficacy and safety of insulin glargine in hyperglycemic acute stroke patients receiving intensive care
Source: Sci Rep. 2021 Jun 1;11:11523. doi: 10.1038/s41598-021-91036-2 (PMC8169927; doi:10.1038/s41598-021-91036-2)
Supplement: Supplementary file 2 — Supplementary Tables. [file 41598_2021_91036_MOESM2_ESM.docx]

Supplementary Table 1. The parameters of acute stroke patients with favorable or non-favorable outcome

|  | mRS ≤ 2  (n=14) | mRS > 2  (n=34) | *P* |
| --- | --- | --- | --- |
| Age, year | 49.2±7.5 | 69.4±13.6 | <0.001* |
| Male (N) | 13 (92.9) | 17 (50.0) | 0.008* |
| BMI (kg/m^2^) | 25.6±4.5 | 28.4±4.2 | 0.049* |
| Weight (kg) | 79.0±13.7 | 66.7±14.8 | 0.010* |
| History of stroke | 3 (21.4) | 10 (29.4) | 0.728 |
| Pre-mRS ≥ 2 | 0 (0) | 8 (23.5) | 0.085 |
| NIHSS at admission | 6 (4, 9) | 16 (11, 22) | <0.001* |
| Atrial fibrillation | 1 (7.1) | 3 (8.8) | 1.00 |
| Diabetes mellitus | 12 (85.7) | 31 (91.2) | 0.621 |
| Hypertension | 11 (78.6) | 28 (82.4) | 1.00 |
| Hyperlipidemia | 6 (42.9) | 13 (38.2) | 1.00 |
| CAD | 5 (35.7) | 6 (17.6) | 0.258 |
| ICH | 7 (50.0) | 5 (14.7) | 0.024* |
| Smoking | 3 (21.4) | 9 (26.5) | 1.00 |
| IV rt-PA | 3 (21.4) | 9 (26.5) | 1.00 |
| EVT | 3 (21.4) | 8 (23.5) | 1.00 |
| SIE | 2 (14.3) | 13 (38.2) | 0.171 |
| TG (mg/dl) | 230.43±131.46 | 185.29±233.83 | 0.502 |
| Cholesterol (mg/dl) | 198.00±56.06 | 177.59±61.25 | 0.288 |
| LDL (mg/dl) | 118.86±30.92 | 102.82±42.11 | 0.205 |
| Glucose at admission (mg/dl) | 311.71±143.39 | 289.00±96.78 | 0.526 |
| HbA1_C_ (%) | 9.70±2.20 | 9.51±1.99 | 0.771 |
| Creatinine (mg/dl) | 1.09±0.35 | 1.10±0.41 | 0.954 |
| Hemoglobin (g/dl) | 15.51±1.58 | 13.77±2.16 | 0.009* |
| Hematocrit (%) | 44.95±4.26 | 41.07±5.92 | 0.031* |
| WBC (k/ul) | 8.87±2.80 | 9.93±2.97 | 0.256 |
| Platelet (k/ul) | 229.86±67.74 | 234.35±58.51 | 0.818 |
| PTT (seconds) | 26.36±2.24 | 25.29±2.46 | 0.169 |
| INR | 1.01±0.19 | 0.96±0.05 | 0.154 |
| Insulin Glargine | 6 (42.9) | 18 (52.9) | 0.752 |
| Glucose (in trial) |  |  |  |
| Pre- random | 279.71±92.80 | 267.29±59.39 | 0.582 |
| 80-180 mg/dl (%) | 55.19±27.54 | 47.41±24.77 | 0.349 |

Data was expressed as number (proportion) or mean± standard deviation.

* indicates statistical significance.

BMI, body mass index; mRS, modified Rankin Scale; NIHSS, National Institute of Health Stroke Scale; CAD, coronary artery disease; ICH, intracerebral hemorrhage; IV, intravenous; EVT: endovascular thrombectomy; SIE: stroke in-evolution; TG, triglyceride; LDL, low-density lipoprotein; Hb_A1C_, glycated hemoglobin; WBC, white blood cell; PTT, partial thromboplastin time; INR, international normalized ratio.

Supplement Table 2. The daily insulin dose

| Total insulin dose (U) | NPH insulin | Insulin Glargine | P-value |
| --- | --- | --- | --- |
| Day 1 | 40.92±17.63 | 43.38±17.59 | 0.623 |
| Day 2 | 46.04±13.04 | 49.35±13.21 | 0.378 |
| Day 3 | 44.00±13.10 | 50.46±14.09 | 0.107 |
| RI dose |  |  |  |
| Day 1 | 33.21±13.55 | 26.12±11.86 | 0.054 |
| Day 2 | 36.21±10.81 | 29.19±9.40 | 0.018* |
| Day 3 | 34.17±10.87 | 29.00±11.27 | 0.113 |
| Basal insulin | NPH | GI |  |
| Day 1 | 7.71±5.32 | 17.27±9.20 | <0.001* |
| Day 2 | 9.83±3.64 | 20.15±6.63 | <0.001* |
| Day 3 | 9.83±3.71 | 21.46±5.38 | <0.001* |

Data was expressed as mean± standard deviation.

* indicates statistical significance.
